# A Public Medical Service for England

**Published:** 1889-07-06

**Authors:** 


### The Hospital

A Weekly Institutional Journal of

Science, Medicine, Nursing, and Philanthropy.

*For Hospitals, Asylums, Medical Practitioners, Students, Nurses, Families, Parishes, Congregations, and the Charitable Public.*

Vol. VI. No. 145.] [July 6, 1889.

### A Public Medical Service for England.

What is a public medical service, and why is one required for England? A public medical service is an organisation which will supply to all persons who cannot pay the ordinary fees of medical men, but who do not wish to accept charitable relief, the most skilled attendance which is available in their neighbourhood, at fixed and strictly limited charges. Such a service is required for England because the respectable working classes are obliged either to avail themselves of inferior skill on account of their inability to pay average fees, or to accept charitable medical relief, which is an injury and a degradation to them. The fact that we are compelled to maintain by argument the right and duty of the British working-man to pay for his own medical attendance exactly as he pays for his coat, his tea, and his house, is one which should give pause to statesmen and political economists, and also to all other persons who may be in any way responsible for the present state of things. That every able-bodied man should in all ordinary circumstances provide for all his own wants, is one of those elementary and self-evident propositions which no person of capable judgment will for a moment call in question. In this particular, of providing for all his own wants, the working-man stands on precisely the same level as every other man. It is as much his duty to do that as it is to pay taxes and obey the laws of his country.

Our claim on behalf of the working classes is one of equality. He who denies that claim is an enemy to the workman. It is only as a man has conceded to him the full rights of citizenship that he rises to its highest duties. In a country governed by popular Government, and influenced by popular ideas the gravest possible responsibility rests upon all classes who help in the establishing of customs or in the dissemination and maintenance of popular ideas. Medical men do not always see this. In many cases the blindest are those who occupy the highest places in the profession. Untrained in the school of statesmanship or of political economy, unaccustomed to reasoning on questions outside the practice of their profession, they often behave like impulsive children when subjects of the most serious import are brought before them for decision. Is it or is it not of importance to the State that its working machinery—its industrial classes—shall be men of honesty, of capacity, of intelligence, and of free and independent character? Can they be men of free and independent character if they are taught from birth to depend for one of the prime necessaries of life—namely, medical attendance—on the charity of their fellow-men, or upon the poor rates? To ask these questions is to answer them. If, as Solomon said, "The borrower is servant to the lender," much more is he who wholly or partly depends upon charity, the servant, the begging slave, of him who provides the charity. It is futile to deny this. Even the very house surgeon acts upon the assumption that the hospital patient is a totally different person from the paying patient, and may be treated with a lofty patronage or an easy off-handedness which he would not think of practising towards any patient outside the hospital.

Nobody who knows the facts will deny that the question of the medical treatment of the working classes has fallen into a state of confusion and uncertainty which is nothing short of disgraceful to those who have brought it about. Take this one short series of facts as an illustration. In 1885, 130 London hospitals treated 1,179,661 out-patients; 18 Liverpool hospitals treated 244,831, and 12 Glasgow charities treated 72,217. The total for these three cities was, therefore, in that year 1,496,709 out-patients. It is said that, tested by a fair wage standard, 50 per cent. of those persons had no right to the benefits of free hospital treatment. That statement is on the whole probably true. At any rate, the circumstances of out-patients have been examined in almost every conceivable way; and the most determined upholders of the *status quo* are compelled to admit that at least 8 per cent. could pay the charges of provident dispensaries. A "public medical service," constituted and carried out on right lines, would take charge of the 50 per cent. of persons who now, without justification, seek hospital aid, would treat them as well or better than hospitals treat them, and would charge them a fixed scale of fees which, though well within their means, would give just and reasonable remuneration to the physicians and surgeons who belonged to the organisation.

It is proposed by a gentleman, Dr. R. R. Rintoul, of Liverpool, who has devoted very careful attention to the question for a number of years, that the medical men of every town and district in England shall combine for the purpose of rendering effective and economical service to the industrial classes, and that those classes themselves shall meet the medical combination by an industrial combination, which shall make it possible for the workers to pay the doctors just and adequate remuneration for their services. The proposal is that the new organisation shall embrace the whole country, so that every member of it, by virtue of his membership, shall receive at any moment the medical aid he needs in whatever part of the country he may happen to be at the time. It will be, in short, a kind of medical freemasonry, which will entitle the initiated to the fullest possible benefits of the organisation under all circumstances.

A brief sketch of the proposed organisation, so far as it is already shaped, will put readers in possession of the main facts. The prime object is that wage-earners whose incomes range between 20s. and 45s. per week for the entire family may secure the best medical attendance by small immediate payments of 2s. for the first visit and 1s. for subsequent visits during the same illness, or 4s. per week paid in advance for five visits. This charge will not cover the cost of drugs. The persons included under the wage limit may belong, not only to the general body of trade operatives, but to the following classes—viz., small shopkeepers, shop and factory hands, poor clerks, domestic servants, and so on. Many of those classes now crowd the out-patient departments of hospitals to their own shame and mortification, and to the grievous burdening of hospital supporters, some of whom are as hard pressed for money as the patients themselves. The rules provide for the division of towns into suitable districts, for the renting of convenient premises, and for the appointment of an adequate number of thoroughly competent physicians, surgeons, and dentists. Each medical man may select that branch of practice in which he is specially proficient. No unqualified man shall be employed in any professional capacity in connection with any branch of the organisation. Certain classes of qualified practitioners will be excluded. The registered chemists in the neighbourhood of each central station will supply medicines at a low fixed scale of charges. The medical staff will, by the constitution of the organisation, have the right to refuse treatment to any who may be outside the 45s. wage limit. Small special fees will be charged for surgical operations, vaccination, and for certain parts of dentistry. Each town and city, in addition to its working staff at the different branches, will have a central board, consisting partly of medical men, dentists, and chemists, and partly of elected laymen. Every member of the organisation will have a card of membership, which, as we have said, will entitle him to the full benefits of the service under all reasonable circumstances.

Such, in briefest outline, is the scheme as it now stands. It does not require more than a moment's thought to enable anyone to see that it is an intelligent effort to deal with a problem which has long been the despair both of hospital managers and rational philanthropists. For its effectual working there will be needed the sympathetic co-operation of the medical profession generally and of hospitals in particular. The weak spots in the proposals are the absence of provision for patients at their own homes and the renting of new premises. But it is probable that the originators intend that the hospitals shall be largely made use of to supplement their organisation. It is impossible at the end of an article to do any kind of justice even to the main outlines of the scheme. We shall, however, return to it at an early opportunity. But what every man should see in it is this: A practical and intelligent proposal which will deal with the whole question and include the whole country; which will raise the working classes out of the slough and mire of a kind of charitable relief which is often as inefficient as it is unnecessary; which will deliver medical men from that insane competition which ruins both their finances and their character at the same time; and which will so lighten the burdens of hospital supporters as to make them at least possible to be endured. It remains to be seen whether members of the medical profession have the brotherly regard for each other and the *esprit de corps* which will enable them to work together for their mutual profit and comfort, and for the undoubted advantage of the wage-earning class.

